# Enhancement of Spin Transport Properties in Angled-Channel Graphene Spin Valves via Hybrid Spin Drift-Diffusion

**DOI:** 10.3390/nano15171367

**Published:** 2025-09-04

**Authors:** Samuel Olson, Kaleb Hood, Otto Zietz, Jun Jiao

**Affiliations:** Department of Mechanical and Materials Engineering, Portland State University, Portland, OR 97201, USA; samolson@pdx.edu (S.O.); kahood@pdx.edu (K.H.);

**Keywords:** graphene, spintronics, non-local spin valve, carrier drift

## Abstract

Graphene has promise as a channel connecting separate units of large-scale spintronic circuits owing to its outstanding theoretical spin transport properties. However, spin transport properties of experimental devices consistently fall short of theoretical estimates due to impacts from the substrate, electrodes, or defects in the graphene itself. In this study, we fabricate both traditional non-local spin valves (NLSVs) and novel hybrid drift-diffusion spin valves (HDDSVs) to explore the impact of charge current and AC spin injection efficiency on spin transport. HDDSVs feature channel branches that allow investigation of charge-based spin drift enhancement compared to diffusion-only configurations. We investigate the modulation of spin transport through hybrid drift-diffusion, observing a decrease in spin signal by 11% for channels with a 45° branch angle, and a 21% increase in spin signal for 135° branch angle channels. We then fabricate symmetrical 90° channel branch angle devices, which do not produce consistent spin transport modulation in drift diffusion mode. These findings highlight the role of carrier drift in enhancing or suppressing spin transport, depending on channel geometry and injection configuration. Overall, our work demonstrates a promising approach to optimizing spin transport in graphene devices by leveraging hybrid drift-diffusion effects without requiring additional DC current sources.

## 1. Introduction

Spintronics is a subfield of electronics which employs the spin of an electron as a degree of freedom instead of its charge [1,2]. Spintronic-based logic has many potential advantages over traditional complementary metal oxide semiconductor (CMOS) architectures, including increased processing speed [3] and lower power draw [4,5,6]. The cornerstone of spintronic logic devices is the ability to inject a current of spin-polarized electrons via a ferromagnetic (FM) electrode which then propagates through a nonmagnetic spin transport channel and is detected by a separate FM electrode. Ideally the spin transport channel will have minimal interference with the electrons as they propagate thus preventing them from relaxing and losing their spin polarization [7,8,9,10].

Graphene, a two-dimensional allotrope of sp^2^-hybridized carbon atoms, has emerged as an ideal candidate for use as a spin transport channel due to its low spin–orbit coupling [11]. The traditional device used for testing the spin transport characteristics of a channel material is the lateral non-local spin valve (NLSV) [12,13]. In a graphene-based NLSV device, pure spin current is injected into the graphene channel via a pair of FM electrodes and diffuses laterally to a second pair of FM electrodes that detect the difference in chemical potential due to the non-equilibrium spin population as a voltage. This non-local voltage is then normalized by the injected current to provide the non-local resistance (R_NL_ = V_det_/I_inj_). The value of R_NL_ depends on the relative orientation of the spins of the electrons and the magnetic polarization of the FM detecting electrodes. The magnetic polarization of the electrodes can be controlled by sweeping an external magnetic field, changing the relative alignment of injecting and detecting electrodes from parallel to anti-parallel. The difference in the value of R_NL_ between the parallel and anti-parallel magnetization states is termed the non-local spin signal (∆R_NL_) and is proportional to the amount of injected spin-polarized current reaching the detecting electrodes. Recording ∆R_NL_ at varying electrode spacings allows for calculation of the spin diffusion length [14,15]. Theoretical calculations estimate a spin diffusion length in graphene of over 100 µm [16]; however, experimental values recorded from graphene-based NLSVs consistently fall short of estimations, with most reports observing lengths less than 10 µm [17,18,19]. One issue that contributes to this discrepancy between experiment and theory is the interface between the FM electrodes and the graphene sheet, which must be a perfect, pin-hole free tunneling barrier in order to obtain high spin injection efficiency without spin-polarized electrons simply re-entering the electrode [20,21,22,23]. Other potential issues include substrate effects [24,25,26], environmental effects [27,28], and grain boundaries in the graphene sheet [29,30]. Improvements to spin diffusion length have been achieved by suspending the graphene channel above the substrate [31], or by encapsulating the graphene channel with hexagonal boron nitride (hBN) [32,33,34]; however, these modifications result in a much more complex manufacturing process. Ingla-Aynés et al. showed that spin diffusion length can be improved in graphene by the addition of a separate DC current to introduce carrier drift for enhanced transport of diffusing spin carriers, resulting in improved spin signal [35].

In this work we study multiple spintronic device modalities fabricated in-house from commercially available graphene on silicon dioxide (SiO_2_). We first systematically characterize the quality and electrical transport properties of the graphene, observing minimal sheet resistance and large areas of monolayer film. We then investigate the basic spin transport properties of the graphene via traditional diffusion-only NLSVs, where we observe spin transport lengths which agree with previously reported values for graphene on SiO_2_. Finally, we introduce novel graphene hybrid drift-diffusion spin valves (HDDSV), which allow for spin transport modulation due to interaction between carrier drift and spin diffusion via the injected AC current without the need for an additional DC current source. This effect is observed by detecting the non-local voltage in a branched channel positioned at varying angles from the main graphene channel where charge and spin carriers are injected. We find that channels with asymmetric branches exhibit modulated spin transport due to the presence of carrier drift in the main channel, while symmetric branched channels with angles of 90° from the main channel show no such consistent deviation from pure spin diffusion. Presence of the hybrid drift-diffusion effect in these devices is confirmed by measuring the difference in spin signal at various charge carrier densities.

## 2. Materials and Methods

### 2.1. Device Design and Fabrication

**Figure 1:**
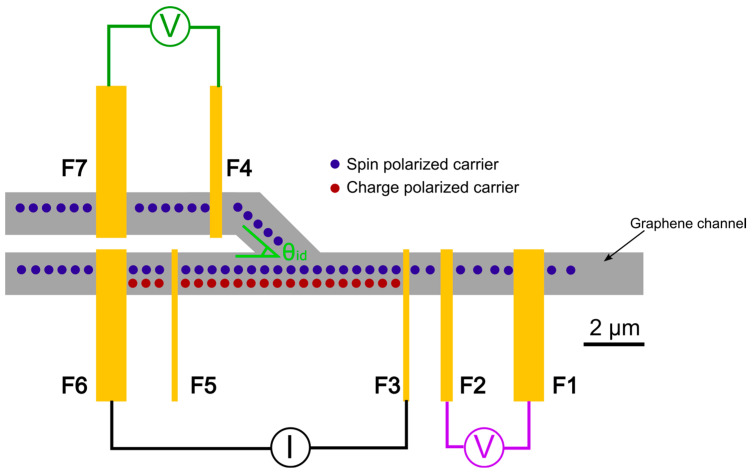
HDDSV layout showing the flow of charge polarized carriers (red dots) and spin polarized carriers (blue dots). The device includes two current injection electrodes (F3, F5), two non-local detection electrodes (F2, F4), and three reference electrodes (F1, F6, F7). The device can operate in standard non-local spin valve mode when the voltage is detected between electrodes F1 and F2 (purple voltmeter), or in hybrid drift diffusion mode when the voltage is detected between electrodes F4 and F7 (green voltmeter). The angle between injector and detector electrodes (θ_id_) can be varied to study the impact of channel angle on spin drift-diffusion.

A schematic of the HDDSV layout is shown in Figure 1. The device consists of a branching graphene channel contacted by seven FM electrodes. The graphene channel is 1.4 µm in width, and the main linear channel has a length of 21 µm. The channel branch departs from the linear portion at an angle of θ_id_ from the main channel. There are two 200 nm wide injector electrodes, F3 and F5, that are used to introduce a spin imbalance into the graphene channel, which results in symmetrical spin diffusion away from the electrode. These are positioned 3.7 µm on either side of the center of the junction in the channel. Additionally, there are two 400 nm wide detector electrodes used for detecting the spin imbalance as a voltage—one on the linear channel (F2), and one on the branched channel (F4). There are also three 1 µm wide reference electrodes (F1, F6, and F7) that can be used either as a drain for the injected current, or for acting as the reference electrode when detecting the non-local voltage. All electrodes are ferromagnetic to simplify the fabrication process. Each electrode type (injector, detector, or reference) has a slightly different width to provide varying magnetic susceptibility when sweeping the external magnetic field during measurements. This electrode layout allows for the ability to take measurements in the standard NLSV mode, as shown in Figure 1 with the purple voltmeter, or HDDSV measurements as shown with the green voltmeter. In the standard NLSV mode, only pure spin diffusion current is detected due to the current injection taking place in an isolated portion of the channel. Current injected through the FM electrode results in a spin imbalance which diffuses down both sides of the channel symmetrically, while charge current is confined to the region containing red dots between the injector and injector reference (electrodes F3 and F6 in Figure 1). In the HDDSV mode, diffusing spin carriers receive a boost from the electric field between the injecting electrodes due to carrier drift. The charge carriers continue down the main channel to the current reference (F6) while diffusing spin carriers can enter the branching channel, as indicated in the region with blue dots. Alternatively, to independently assess the impact of the angled channel on spin transport, voltage can be detected in the branched channel while current is injected between electrodes F3 and F1, effectively preventing the charge carriers from interacting with spin carriers diffusing toward the branch junction. This arrangement allows for the study of AC spin drift diffusion effects in branched channels without the need for an additional set of electrodes for introducing a DC current.

The devices were fabricated using a simple 3-layer process. The starting material was a 4″ graphene-on-SiO_2_ wafer sourced from Grolltex Inc., San Diego, CA, USA. The wafer was cleaned in isopropyl alcohol and acetone and then coated with a resist of poly methyl methacrylate (PMMA). The first layer (alignment markers) was patterned via electron beam lithography (EBL) followed by development and electron-beam deposition of 5 nm Ti, 50 nm Au. The second layer (graphene channel definition) was again patterned via EBL followed by a graphene oxygen plasma etch (50 W, 5 mTorr, 60 s). The third layer (FM electrodes) was patterned via EBL followed by electron beam deposition of 1 nm Ti. This Ti layer was oxidized by flooding the deposition chamber with pure oxygen to a pressure of 1 Torr and holding for 30 min. This was followed by deposition of a further 1 nm of Ti and subsequent oxidization for a total tunnel barrier thickness of 2 nm. On top of the tunnel barrier, 25 nm of Co was deposited via electron beam deposition to form the FM electrodes. Metal lift-off was performed in warm acetone, and the device array was then placed in a probe station under vacuum for testing to avoid further oxidation of the electrodes.

### 2.2. Device Characterization

Electrical, structural, and spin transport properties of the devices were assessed to fully characterize the HDDSV design. Electrical characterization and spin transport measurements were conducted under vacuum (~10^−6^ Torr) at room temperature in a Lakeshore magnetic probe station. Transfer length method (TLM) measurements were performed using an Agilent B1500A semiconductor parameter analyzer (Agilent Technologies, Santa Rosa, CA, USA). Charge neutrality point measurements were accomplished using the P-doped Si wafer as a backgate, where the gate voltage was swept between −80 V and +80 V using the Agilent B1500A through the chuck of the probe station. For spin valve measurements, a 10 µA current with a 13 Hz AC carrier wave was injected into the devices via a Keithley 6221a current source (Keithley Instruments, Portland, OR, USA), and the non-local voltage was detected with a Stanford Research Systems SR850 lock-in amplifier (Stanford Research Systems, Inc., Sunnyvale, CA, USA) while the probe station’s parallel electromagnet was swept between magnetic field strengths of −50 mT to +50 mT. Optical images were taken on a Nikon Eclipse L200 microscope (Nikon Instruments Inc., Tokyo, Japan) at 50× magnification, and SEM micrographs were obtained using an SEC SNE-4500M Plus (SEC Co., Ltd., Suwon-si, Republic of Korea). Raman spectra were acquired using a Horiba HR800 UV spectrometer (Horiba Ltd., Kyoto, Japan) with a 100 mW, 532 nm excitation laser.

## 3. Results and Discussion

### 3.1. Graphene Quality

**Figure 2:**
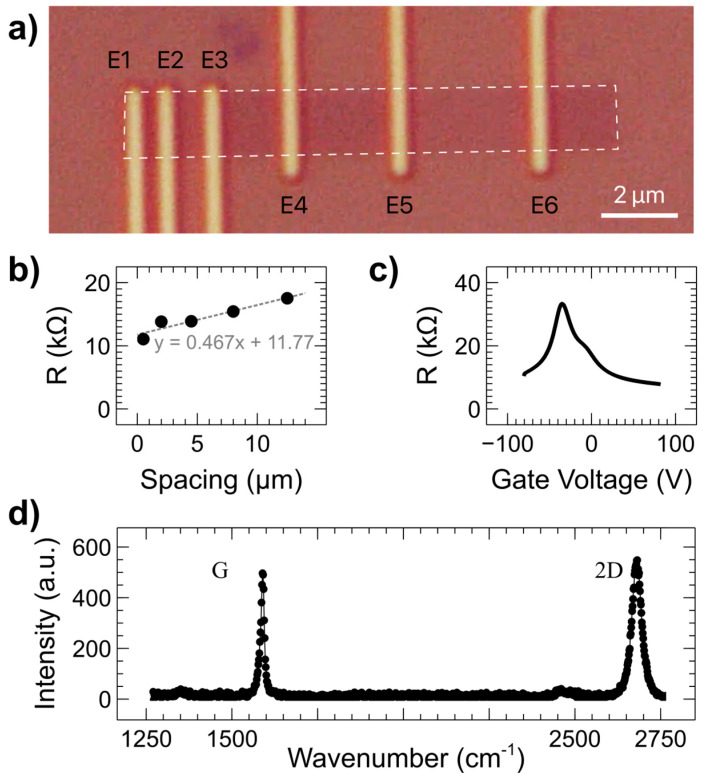
Analysis of electrical and structural quality of the graphene film. (**a**) Optical image of transfer length method (TLM) measurement device (the graphene channel is outlined with a white dashed line for enhanced visibility), (**b**) TLM measurement with linear fit showing sheet resistance *R_S_* = 930 Ω and contact resistance *R_C_* = 5.9 kΩ, (**c**) backgate voltage sweep between electrodes E1 and E6, (**d**) representative Raman spectrum of graphene from device area with G and 2D peaks labeled.

The devices were fabricated from commercially available graphene that was grown via chemical vapor deposition and subsequently transferred onto an SiO_2_ substrate. A transfer length measurement (TLM) device was used to determine basic electronic properties of the graphene (Figure 2a). This device employs electrodes at intervals of increasing length to extract the sheet resistance (*R_S_*) and the contact resistance (*R_C_*) of a sample [36]. In this case, the TLM electrodes are the same as the FM HDDSV electrodes (25 nm Co on top of 2 nm TiO_2_). Current versus voltage curves were taken between electrodes E1 and E2–E6, and resistance was determined by the resulting slope. The total resistance versus electrode spacing is shown in Figure 2b, with a linear fit to the data and its equation shown in gray. The fit corresponds to the equation:

(1) $$\begin{array}{c} R_{tot}=\frac{R_{S}}{W_{ch}}x+2R_{C}, \end{array}$$

where *W_ch_* = 2 µm is the TLM channel width, and *x* is the electrode spacing. From the linear fit equation, it is straightforward to determine that *R_S_* = 930 Ω and *R_C_* = 5.9 kΩ. The value of *R_C_* indicates that the contacts are somewhere between transparent and tunneling likely due to pinholes in the tunnel barrier layer—a common issue with insulating tunnel barrier contacts [37]. The resistance between electrodes E1 and E6 versus backgate voltage is shown in Figure 2c, with the peak at −35 V representing the charge neutrality point where charge carrier density is lowest. The dominant carriers are electrons (holes) at backgate voltages more positive (negative) than this point. The deviation of the backgate voltage from the theoretical 0 V point and the asymmetric shape indicate that there is some doping of the graphene channel, potentially from contaminants on top of the graphene or from substrate effects. A Raman spectrum taken on the graphene channel of a device is shown in Figure 2d. The ratio of the height of the 2D peak (at ~2700 cm^−1^) to the height of the G peak (at ~1600 cm^−1^) is ~1, indicating that the graphene is monolayer [38]. There is no prominent D peak (typically found at ~1350 cm^−1^), indicating that the graphene has a very low defect density.

### 3.2. 45° Hybrid Drift-Diffusion Spin Valves

**Figure 3:**
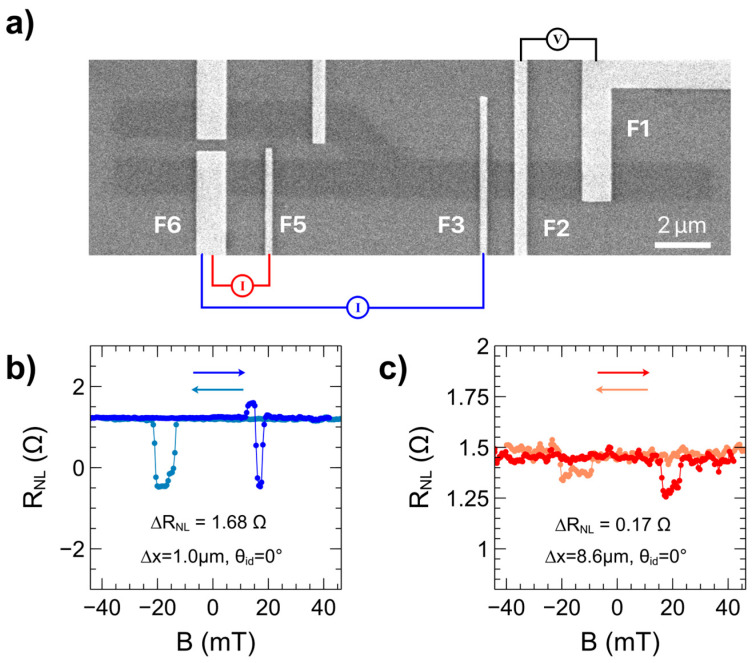
Spin transport in standard NLSV mode. (**a**) SEM micrograph of standard non-local spin valve electrode arrangements, (**b**) non-local voltage versus parallel magnetic field sweep for ∆x = 1.0 µm, θ_id_ = 0° (current injected between electrodes F3 and F6, voltage detected between electrodes F2 and F1) and (**c**) ∆x = 8.6 µm, θ_id_ = 0° (current injected between electrodes F5 and F6, voltage detected between electrodes F2 and F1). Darker traces represent forward sweeps (increasing magnetic field strengths), lighter traces represent reverse sweeps (decreasing magnetic field strengths).

The HDDSV devices have the ability to operate in standard NLSV mode to determine baseline spin transport properties. Electrode configurations for NLSV mode are illustrated in Figure 3a. In this mode, the current injection occurs away from the detecting contacts, resulting in a non-local spin signal due to diffusion of spin current alone. The channel is linear and so the channel branch angle between injector and detector electrodes (θ_id_) is zero. The electrode configuration and non-local resistance versus magnetic field for the injector electrode F3 and detector electrode F2 configuration are shown in Figure 3b. In this configuration, AC charge current is injected between electrodes F3 and F6, and F2 and F1 act as the detector and detector reference respectively. Charge carriers will be restricted to the region between F3 and F6, while spin carriers will diffuse out from F3 in both directions. A strong spin signal of 1.68 Ω is observed due to the small injector-detector spacing (∆x) of 1.0 µm—over this short distance, most of the spin carriers maintain their initial spin polarization. When this distance is increased to 8.6 µm, as shown for the electrode configuration in Figure 3c where spins are injected via electrode F5, the spin signal drops an order of magnitude to 0.17 Ω. Using the spin signal values at varying electrode spacings, the spin diffusion length (*λ*) can be calculated using the below formula [39]:

(2) $$\begin{array}{c} \ln\left(\Delta R_{NL}\right)=-\frac{1}{\lambda }L+\ln\left(\frac{1}{\sigma }\frac{P_{j}^{2}\lambda }{W}\right)\;\;, \end{array}$$

where *σ* is the conductivity of graphene, *P_j_* is the spin injection efficiency, *W* is the width of the graphene channel, *L* is the electrode spacing, and *λ* is the spin diffusion length. By plotting the spin signal versus electrode spacing on a log-log plot, the spin diffusion length can be obtained as the negative inverse slope of the linear fit. For these devices, the spin diffusion length was found to be approximately 3.3 µm. This is likely an underestimate, as the presence of pinholes in the F3 electrode, between electrodes F5 and F2 in Figure 3c, could also contribute to spin relaxation, shorten the spin relaxation time, and thus reduce the spin diffusion length.

**Figure 4:**
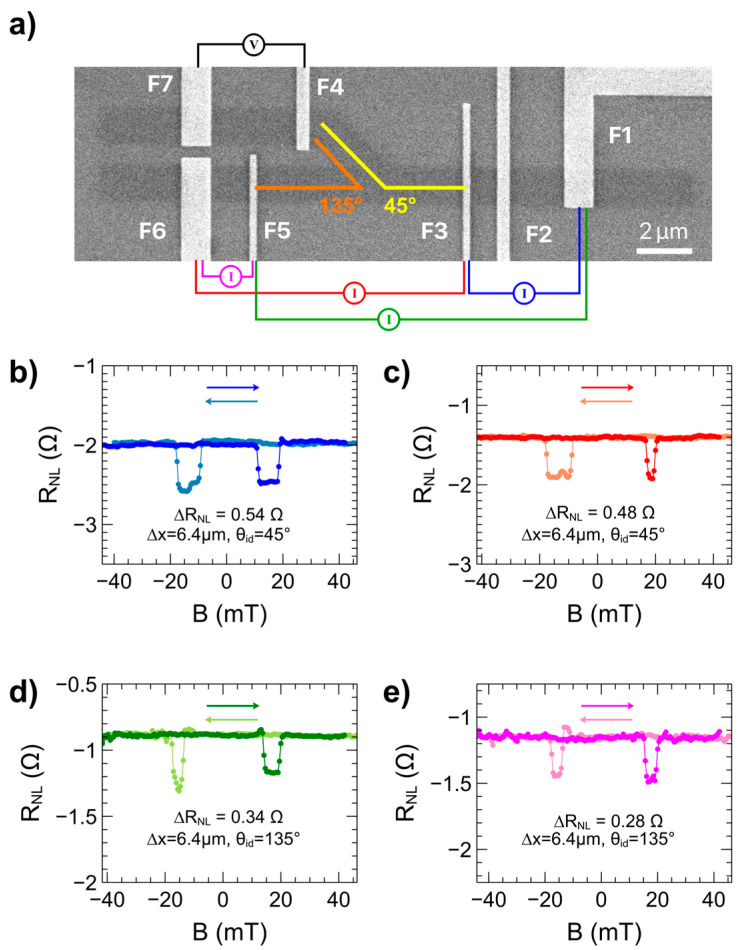
Spin transport in HDDSV mode. (**a**) SEM micrograph of electrode arrangements for HDDSV mode. Corresponding non-local voltage versus parallel magnetic field sweeps for (**b**) ∆x = 6.4 µm, θ_id_ = 45°, current injected between electrodes F3 and F1, (**c**) ∆x = 6.4 µm, θ_id_ = 45°, current injected through electrodes F3 and F6, (**d**) ∆x = 6.4 µm, θ_id_ = 135°, current injected between electrodes F5 and F1, (**e**) ∆x = 6.4 µm, θ_id_ = 135°, current injected between electrodes F5 and F6. Electrodes F4 and F7 acted as the non-local voltage detectors in each sweep. Darker traces represent forward sweeps (increasing magnetic field strengths), lighter traces represent reverse sweeps (decreasing magnetic field strengths).

Once NLSV spintronic measurements were completed, the HDDSV mode of the devices in the branched channel was investigated. Prior studies have demonstrated the ability to detect diffusing spin carriers in branched channels on CVD graphene; however, those devices operated in a pure diffusion mode [40]. Here we investigate the ability of the channel branch to act as an isolated spin detector which can be modulated by charge carrier drift. Figure 4 provides data of magnetic sweeps wherein the spin current is detected as a voltage in the branched channel of the devices (between electrodes F4 and F7). The electrode arrangements possible in HDDSV mode are outlined in Figure 4a, where spins are injected through either electrode F3 or F5 with an AC current, drained to either electrode F1 or F6, and detected as a voltage between electrodes F4 and F7. Figure 4b,c show magnetic sweep data for the case where θ_id_ = 45°, with current injected from electrode F3. In Figure 4b, the current injection loop exists between the injector electrode F3 and the drain electrode F1 (blue current source in Figure 4a). In this configuration, the branched detector is completely outside of the current injection loop and the voltage detected is due entirely to spin diffusion resulting in a spin signal of 0.54 Ω. When the injection current drain electrode is switched to F6, as in Figure 4c, the detected spin signal drops by 11% to 0.48 Ω. In this mode, charge carriers moving between the injector electrode (F3) and the drain electrode (F6) pass the channel branch. It is hypothesized that this significant decrease in spin signal is due to a small amount of drift taking place in the graphene channel, acting against the diffusing spin polarized carriers.

The design of the HDDSV device enables two effective injector-detector angles on a single device owing to the placement of injector electrodes on either side of the channel branch. This configuration facilitates the study of the impact of channel angle while keeping the channel material and electrode properties consistent. In Figure 4d,e, the current injecting electrode is F5 and θ_id_ = 135°. In Figure 4d, the injected current is drained at electrode F1 (green current source in Figure 4a). The channel branch lies within the current injection loop and a spin signal of 0.34 Ω is observed. When the drain electrode is switched to F6 as in Figure 4e, the spin is diffusion-based and the detected spin signal drops by 18% to 0.28 Ω (magenta current source in Figure 4a). The observation that the configuration with the channel branch within the current injection loop causes a lower spin signal than pure spin diffusion for θ_id_ = 45° and a higher spin signal than pure spin diffusion for θ_id_ = 135° can be understood by considering the relative placement of the injecting electrode with respect to the channel branch. A plausible explanation for the differences in spin signal between the two injector-drain modalities is the presence of carrier drift within the channel, which may result from a small DC offset in the injected AC current, as well as the asymmetry of the channel branch. In the case of Figure 4c, the diffusing spin carriers must travel against the electric field-induced drift as they traverse from the drain electrode F6 to the injecting electrode F3, opposite the direction of the detectors on the branching channel. This results in a lower spin signal compared to the diffusion-only modality in Figure 4b. In contrast, in Figure 4d the diffusing spin carriers are traveling in the same direction as the drift set up by the electric field between electrodes F1 and F5, leading to an enhanced spin signal relative to the diffusion-only modality in Figure 4e. The asymmetry of the channel branch further contributes to a net drift effect that influences the diffusing carriers. As carriers with drift-modulated momentum in the main channel enter the branch during the “positive” half-cycle of the AC current, they become less susceptible to opposing (negative) drift during the polarity reversal of the “negative” half-cycle. This temporal asymmetry may enhance net spin accumulation in one direction over a full AC cycle.

**Figure 5:**
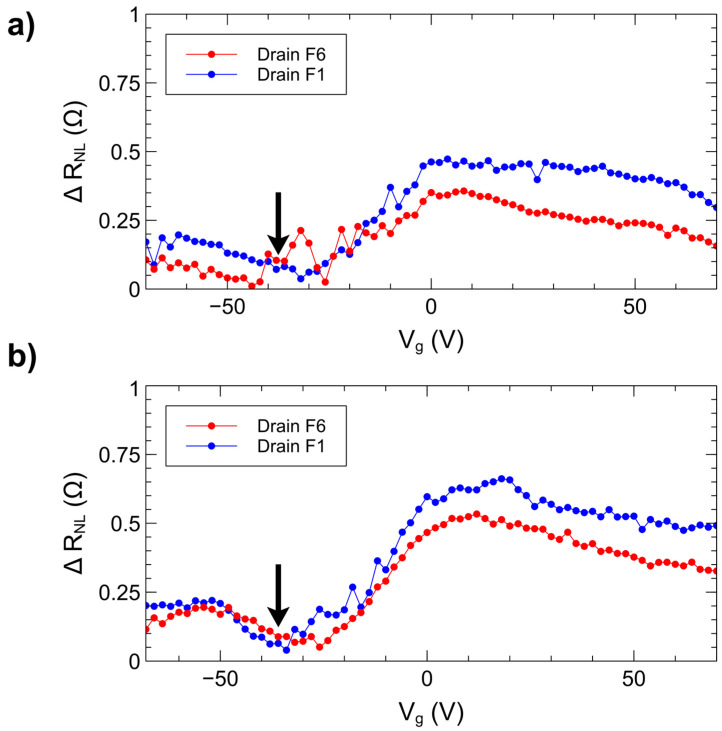
Non-local spin signal versus backgate voltage for current injected through electrode. (**a**) F3 and (**b**) F5. Black arrows indicate the position of the charge neutrality point.

The presence of the hybrid drift-diffusion effect can be further investigated by observing the change in non-local spin signal as the charge carrier density is varied, which can be accomplished in graphene on SiO_2_ by applying a voltage to the highly doped silicon backgate [41]. Figure 5 shows data for ∆R_NL_ versus backgate voltage (V_g_) when current is injected through electrode F3 (Figure 5a) or electrode F5 (Figure 5b) and drained to reference electrode F6 (red curves) or reference electrode F1 (blue curves). For each point in these measurements, the backgate was held at a specific voltage while a magnetic field sweep was performed. The resulting spin signal is then plotted versus V_g_. The charge neutrality point is indicated by the black arrow (−35 V, as shown in Figure 2c). At backgate voltages more negative than the charge neutrality point, the majority charge carriers are holes while at more positive voltages they are electrons. For both injector electrodes F3 and F5, the difference in spin signal obtained between the two reference electrodes F1 and F6 is considerable in the electron-dominant region. Near the charge neutrality point, the charge carrier density is at its lowest, and the difference between the two references for both injector electrodes is minimal. At backgate voltages below −35 V, the difference in spin signal again begins to increase, indicating that a similar effect is observed when the dominant carriers are holes. The minimal difference in spin signal at the charge neutrality point indicates that the change in spin signal magnitude in hybrid drift-diffusion mode is due in part to the presence of higher concentrations of charge carriers.

### 3.3. 90° Hybrid Drift-Diffusion Spin Valves

**Figure 6:**
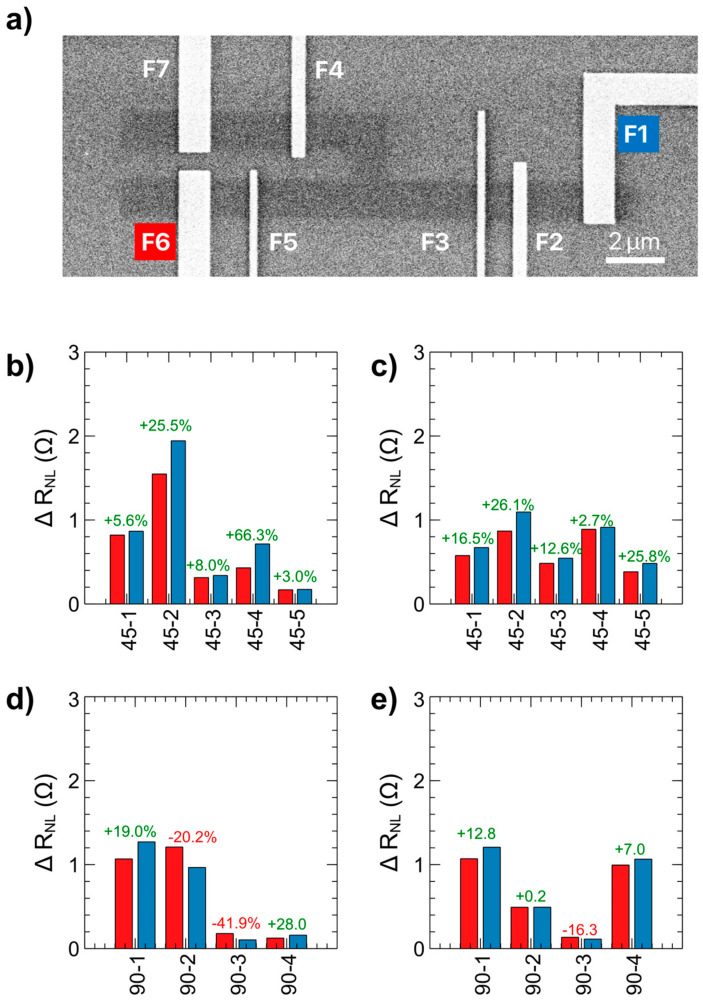
(**a**) SEM micrograph of a 90° channel angle HDDSV with electrodes labeled. (**b**,**c**) Spin signal values for 45° channel angle devices with current injected through F3 and F5 respectively. (**d**,**e**) Spin signal values for 90° channel angle devices with current injected through F3 and F5 respectively. Red bars indicate that current is drained at the F6 reference electrode, blue bars indicate that current is drained at the F1 reference electrode. ∆x = 6.4 µm for all data points.

Dependence of the hybrid spin drift-diffusion transport mechanism on channel angle was investigated by fabricating HDDSV devices with a 90° branch, as seen in Figure 6a. Here there are again two injector electrodes (F3 and F5) on either side of the branch. Additionally, there are two detector electrodes—one on the linear channel (F2) and one on the branched channel (F4). There is a reference electrode placed at the end of each channel section (F1, F6, F7). This electrode configuration is identical to that of the 45° branched HDDSV as shown in Figure 1, allowing for direct comparison with that device architecture. Spin signal summaries for the 45° branched devices are shown in Figure 6b,c. In the Figure 6b data, the current is injected through electrode F3 and drained to either electrode F6 (red bars) or electrode F1 (blue bars). In Figure 6c, the current is injected through electrode F5. For both injection electrodes on the 45° channel devices, there is a consistently higher spin signal when the injection is drained to electrode F1 due to hybrid-drift-diffusion effects, as explained in Figure 4. However, when the branch angle is increased to 90° as in Figure 6d (current injected through F3) and Figure 6e (current injected through F5), there is not a consistent electrode configuration which provides higher spin signal. For these devices, the average difference between spin signal when current is drained to F1 and F6 is nearly zero. We attribute variation of spin signal magnitude among identical electrode arrangements to inhomogeneities in the CVD graphene structure and inconsistent pinholes in the electrode tunnel barriers. This dependence of the hybrid spin drift-diffusion effect on channel angle can be understood when considering the effect of the electric field drift on the charge carriers as they traverse down the linear portion of the channel. In the branched portion of the channel, the effect of the drift parallel to the linear channel is proportional to cos(θ_id_). As the angle is increased, the effect of the carrier drift momentum on spin transport in the branched channel is reduced. For the 90° channel angle device architecture, the carrier drift-diffusion effect is minimal due to the drift momentum being orthogonal to the channel branch. This results in spin transport in the branched channel taking place mainly via diffusion regardless of whether carrier drift is present in the main channel. This is in contrast to the 45°/135° devices, where the spin signal in the branched channel differs based on whether or not carrier drift is occurring in the main channel, demonstrating the ability to control spin transport based on the channel branch angle.

## 4. Conclusions

In this work, we introduce novel graphene hybrid drift-diffusion spin valves (HDDSVs) and demonstrate their potential to significantly enhance spin transport compared to traditional non-local spin valves (NLSVs), which rely solely on diffusion. Utilizing wafer-scale graphene on SiO_2_ and standard semiconductor fabrication techniques, we fabricated and systematically characterized both NLSVs and HDDSVs, enabling a direct comparison between pure diffusion-based and hybrid drift-diffusion-based transport. Our results reveal that asymmetrical 45° branched HDDSVs exhibit a substantial increase in spin signal when operating in hybrid drift-diffusion mode, whereas symmetrical 90° branched devices do not show consistent enhancement. This spin transport modulation is attributed to the combined effects of drift and diffusion, wherein spin-polarized carriers gain additional momentum from the AC electric field, effectively increasing their spin diffusion length and enhancing the overall spin signal. The observed dependence on charge carrier density—modulated via the back-gate voltage—further supports the role of carrier drift in this mechanism. These findings offer a novel approach to enhancing spin transport in graphene-based devices, with important implications for optimizing large-scale spintronic circuits. The ability to modulate spin transport using hybrid drift-diffusion effects—without requiring additional DC current sources—opens new pathways for improving the performance and scalability of graphene spintronic technologies.

Building on these results, future studies will investigate the influence of an applied DC bias to the AC injection current to better understand how drift magnitude and direction affect hybrid drift-diffusion enhancement. Furthermore, HDDSVs with additional channel angles between 45° and 90° will be fabricated and characterized to gain deeper insight into the impact of channel angle on the underlying drift-diffusion momentum phenomena. These efforts are expected to inform the design of next-generation high-performance graphene spintronic devices.

## Data Availability

Data are contained within the article.
